# Defining the minimally effective dose and schedule for parenteral hydrogen sulfide: long-term benefits in a rat model of hindlimb ischemia

**DOI:** 10.1186/s13618-015-0027-1

**Published:** 2015-04-16

**Authors:** John William Langston, Christopher F Toombs

**Affiliations:** Faraday Pharmaceuticals, Inc., Seattle, WA USA

## Abstract

**Background:**

Peripheral arterial disease (PAD) affects millions of Americans and leads to critical limb ischemia (CLI) in the most severe cases. Investigators have demonstrated the utility of hydrogen sulfide for restoring perfusion in rodent models of chronic ischemia. We sought to determine the minimum effective dose (MED) of sulfide necessary to restore perfusion in the rat hindlimb, to assess the persistence of limb perfusion after cessation of treatment, and to compare perfusion measurements between laser doppler and ultrasound methods.

**Methods:**

In 3 separate experiments, sodium sulfide (1.0, 0.5, or 0.25 mg/kg twice daily for 14 days, 0.25 mg/kg twice daily for 7 days, 0.5 mg/kg once daily for 7 days, or 0.25 mg/kg twice daily for 3 days) or vehicle was administered after left femoral artery ligation and transection. Hindlimb perfusion was assessed by laser doppler flowmetry and contrast enhanced ultrasound over the duration of each study, and cellular proliferation and vascular density were assessed by immunohistochemical means in the initial experiment.

**Results:**

Intravenous sodium sulfide at 0.25, 0.5, or 1.0 mg/kg twice daily for 2 weeks significantly enhanced the recovery of blood flow to the ischemic hindlimb by 7 days. The enhancement of blood flow with 1.0 mg/kg dosing was coincident with an increase in cellular proliferation and vascular density in the ischemic tissue. In a final experiment, i.v. administration of sodium sulfide at 0.5 mg/kg once daily for 7 days or 0.25 mg/kg twice daily for 7 days significantly elevated blood flow and skeletal muscle perfusion in the ischemic hindlimb, whereas 0.25 mg/kg twice daily for 3 days had no effect. This enhancement of blood flow appeared long lived, as blood flow remained elevated 3 weeks after cessation of treatment.

**Conclusions:**

These data, together with other published observations, demonstrate the efficacy of hydrogen sulfide in restoring perfusion to chronically ischemic tissue and establish a minimum efficacious dose in the rat hindlimb model.

## Background

Peripheral arterial disease (PAD) is characterized by a narrowing of the arteries in peripheral vessels caused by atherosclerotic plaque formation, resulting in decreased blood flow to distal appendages. This disorder affects 8 to 12 million Americans and represents a significant financial health care burden that will rise concomitantly with the rates of obesity and diabetes [1]. Critical limb ischemia (CLI), characterized by pain at rest, ulcerations, and/or gangrene in the extremity, is considered an end stage of PAD, with the majority of these patients undergoing revascularization (bypass or angioplasty) or amputation as long as they can tolerate surgery [2,3]. Currently, there is no established nonsurgical treatment regimen for CLI patients.

Hydrogen sulfide (H_2_S) plays a number of important roles in cardiovascular physiology as a regulator of inflammation, angiogenesis, vessel tone, and redox potential [4-6]. It has also been shown to be protective in animal models of cardiovascular disease including heart failure [7-9], hypertension [10,11], atherosclerosis [12,13], and ischemia reperfusion injury in multiple organs and tissues [14,15]. Mechanistically, sulfide is thought to mediate these effects in a variety of ways. As a second messenger and signaling molecule, sulfide is thought to modulate cellular function via reversible cysteine s-sulfhydration of a number of enzymes, transcription factors, and cytoskeletal elements [16-18]. Another purported cytoprotective mechanism stems from observations that sulfide at lower concentrations can reversibly reduce the oxygen affinity of cytochrome c oxidase, effectively dampening cellular respiration [19,20]. The nucleophilic nature of H_2_S is also thought to contribute to its observed efficacy via the attenuation of oxidative stress and reactive oxygen species (ROS) signaling [21].

Therapeutic angiogenesis refers to the pharmacologic induction of neovascularization to restore blood flow and oxygenation in ischemic tissue, and published observations suggest hydrogen sulfide could be such a pharmacophore. H_2_S stimulates endothelial cell migration, proliferation, and tube formation in vitro, and has been shown to stimulate vascular growth in the chicken chorioallantoic membrane and into matrigel plugs implanted in vivo in mice [5,22]. Furthermore, hydrogen sulfide has been shown to stimulate vascular growth in ischemic heart tissue and improve cardiac function in rodent models of heart failure [8,23]. The molecular mechanisms underlying these effects are being investigated, and evidence suggests that H_2_S enhances nitric oxide production and bioavailability [24,25], increases HIF-1α mediated gene expression (including VEGF and its receptors) [24,26], and can activate VEGFR2 directly via reduction of a vicinal disulfide bond [27].

To date, several investigators have demonstrated the capacity of hydrogen sulfide to revascularize and restore blood flow to skeletal muscle in rodent models of hindlimb ischemia (HLI). Wang and colleagues used fluorescent microspheres to assess the effect of NaHS on rat hindlimb perfusion, and Bir et al. used laser doppler flowmetry to demonstrate the beneficial effect of Na_2_S on perfusion of the mouse hindlimb [24,28]. Both studies provided evidence suggesting that H_2_S mediates these proangiogenic effects via the upregulation of VEGF signaling. In the current study, we sought to determine a minimum dose and schedule of parenteral hydrogen sulfide sufficient to restore perfusion in the rat hindlimb. Utilizing contrast enhanced ultrasound to corroborate laser doppler flowmetry results, we demonstrate here that a twice daily intravenous infusion of 0.25 mg/kg sodium sulfide over as few as 7 days was sufficient to significantly enhance perfusion in the ischemic hindlimb. Moreover, the effect on perfusion persisted several weeks after cessation of treatment.

## Methods

### Animals

Male CD rats, 350–450 g with jugular vein catheters (JVC), were purchased from Charles River (Wilmington, MA). All experiments were approved by the internal Ethical Committee for Animal Care and Use. All animals were singly housed and allowed to acclimate in house for at least 48 hours prior to study entry. The facility was kept at a temperature of 21°C, humidity of 45-55%, under a 12 hour light/dark cycle. Animals had access to food and water *ad libitum*.

### Surgical model

Under 1.5% isoflurane, animal fur was removed from the distal hind limbs using depilatory cream, and blood flow was measured as described below. An incision was made in the left groin, and the femoral artery was carefully dissected away from the femoral vein, femoral nerve, and surrounding fascia. The femoral artery was ligated at two points with silk suture just distal to the inguinal ligament, and the vessel was then transected between the two ligation points. The incision in the groin was stapled closed, and hind limb blood flow was measured again as described below.

### Laser doppler flowmetry

A laser doppler flow meter (Product# ML191, ADInstruments, Colorado Springs, CO) was used in conjunction with either a miniature surface flow probe (Product# MSP300XP, ADInstruments, Colorado Springs, CO) or a fine needle flow probe (Product# MNP110XP, ADInstruments, Colorado Springs, CO) to measure hind limb blood flow. Blood flow was recorded and analyzed using a PowerLab 8/30 (Product# ML870/P, Colorado Springs, CO) with LabChart software, and a heat lamp was used to maintain core body temperature while all blood flow measurements were obtained. While under 1.5 % isoflurane, flow measurements were taken from the proximal, medial, and distal portions of each calf twice, for a total of 6 measurements per calf that were averaged to give a single value. Blood flow in the ischemic (left) limb was normalized to blood flow in the nonischemic (right) limb.

### Contrast enhanced ultrasound measurements of hindlimb perfusion

A Vevo 770 high-resolution ultrasound imaging system (VisualSonics, Toronto, Ontario, Canada) equipped with a 15–45 MHz RMV scanhead (Model# RMV707B, VisualSonics, Toronto, Ontario, Canada) was used to visualize the gastrocnemius and measure perfusion within it. While under 1.5% isoflurane, a lateral section of each gastrocnemius parallel to the fibula was visualized. Once a satisfactory image was obtained, 150 μL of a contrast microbubble solution containing 3.0 x 10^8^ microbubbles were given as a bolus via the jugular vein catheter, and the change in contrast visualized within each gastrocnemius muscle was used to indicate perfusion in the ischemic hindlimb relative to the nonischemic limb. A heat lamp and heated stage were used to maintain core temperature of each animal over the course of measurements.

### Immunohistochemical assessment of vascular density and cellular proliferation

Upon completion of the experimental protocol, each animal was euthanized by exsanguination under 3.0% isoflurane anesthesia. An incision was then made in the left ventricle, and 10% buffered neutral formalin was pushed through the lower torso via the abdominal aorta just proximal to the iliac bifurcation. The left (ischemic) and right (nonischemic) gastrocnemius muscles were then excised and placed in 10% buffered neutral formalin, at which point they were taken to the pathology department at the University of Washington for paraffin embedding, sectioning, and staining.

Six short axis slices of each muscle were embedded into paraffin blocks. For vascular density determination, three sections from each block were stained for CD-31 (PECAM-1), an endothelial specific antigen, and counterstained with hematoxylin. Digital images of 10 high power fields within each section were obtained, and the ratio of PECAM-1 positive vessels to myofibers was quantified for each field.

For determination of cellular proliferation, 3 sections from each block were stained for Ki-67, a nuclear antigen expressed only in proliferating cells. Digital images of 10 high power fields within each section were obtained, and the number of Ki-67 positive nuclei per high power field were determined.

### Data analysis

All data presented are expressed as the mean + SEM, and all groups were compared using a one way ANOVA with a Bonferroni post hoc test. P < 0.05 was considered statistically significant.

## Results

### Experiment 1

**Figure 1:**
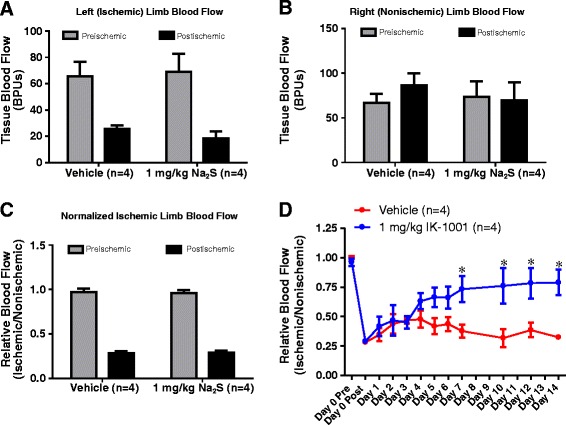
Na_2_S Enhances Blood Flow to the Ischemic Hind Limb. Panels **A** and **B** are laser doppler blood flow values obtained from the ischemic and nonischemic hindlimbs, respectively, of rats before and after femoral artery ligation. Panel **C** illustrates changes in blood flow to the ischemic hindlimb minutes after femoral artery ligation, expressed as the ratio of blood flow measured in the ischemic limb (Panel A) vs. the nonischemic limb (Panel B). Panel **D** illustrates the effect of i.v. administration of Na_2_S (1 mg/kg, twice daily) on blood flow in the ischemic hindlimb. * p < 0.05 vs Vehicle at respective time point by One Way ANOVA. Values are mean ± SEM.

In a pilot experiment, four animals were administered sodium sulfide at 1.0 mg/kg (as a one minute i.v. infusion) twice daily for 14 days, while another 4 animals received vehicle (0.9% sodium chloride) at an identical volume twice daily over the same time period. Figure 1 illustrates the effect of femoral artery ligation on hindlimb blood flow measured by laser doppler flowmetry. Panels A and B illustrate the absolute laser doppler blood flow values obtained in the ischemic and nonischemic hindlimbs, respectively, both before and after femoral artery ligation. Panel C compares blood flow in the left hindlimbs before and after left femoral artery ligation in both treatment groups. In this graph, blood flow in the ischemic limb is expressed as a ratio of flow in the nonischemic (right) hind limb. Prior to the onset of ischemia, blood flow in both limbs was comparable (vehicle - 0.97 ± 0.041 vs 1.0 mg/kg sodium sulfide - 0.962 ± 0.033). In the minutes following femoral artery transection, blood flow was reduced in the ischemic limb to approximately 30% of the preischemic value (vehicle – 0.282 ± 0.023 vs 1.0 mg/kg Na_2_S – 0.290 ± 0.021). Together, these data demonstrate that the decrease in relative blood flow after the induction of ischemia was strictly due to a decrease in blood flow in the ischemic limb; while blood flow in the nonischemic limb was unaffected.

Panel D of Figure 1 illustrates the effect of i.v. administration of Na_2_S on blood flow recovery in the ischemic hind limb. Sodium sulfide administered at 1.0 mg/kg twice daily significantly elevated blood flow in the ischemic limb by day 7 compared to vehicle treatment and was maintained through day 14. These data are consistent with published results from Bir et al. demonstrating an enhancement of blood flow by day 7 in mice administered sodium sulfide at 1.0 mg/kg twice daily [3].

**Figure 2:**
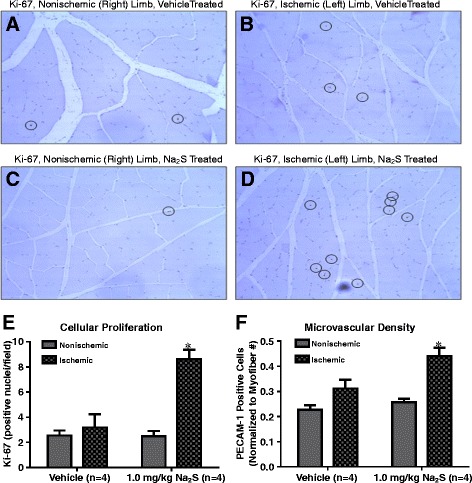
Na_2_S Enhances Cellular Proliferation and Vascular Density in the Ischemic Hind Limb. Panels **A** and **B** are representative images of sections with Ki-67-positive nuclei (circled) from nonischemic and ischemic limbs, respectively, of vehicle treated rats. Panels **C** and **D** are representative images from sections of nonischemic and ischemic limbs, respectively, of 1.0 mg/kg Na_2_S treated animals. Panels **E** and **F** are graphs comparing the average number of Ki-67 positive nuclei and PECAM-1 stained vessels for gastrocnemius muscle across treatment groups. * p < 0.05 vs Nonischemic of the same treatment group by one way ANOVA. Values are mean ± SEM.

After 14 days of treatment, the left and right gastrocnemius muscles from each animal were excised and processed for immunohistochemical assessment of cellular proliferation and vascular density within the tissue. Figure 2 demonstrates the effect of sulfide administration on vascular density and cellular proliferation within the ischemic tissue. In animals receiving a 1.0 mg/kg dose for 14 days, the number of Ki-67 positive nuclei were significantly elevated in the ischemic limb (panels D and E) compared to the nonischemic limb (panels C and E); an effect which was not observed in vehicle treated mice (panels A, B, and E). Similarly, the number of CD-31 positive cells were increased in the ischemic limbs of sulfide treated rats, whereas there was no effect on vascular cell density observed in vehicle treated mice (Panel F).

### Experiment 2

A second study addressed the questions of dose response and minimal effective dosing of sulfide in the model. To these ends, 4 groups of 7 animals received either 1.0 mg/kg, 0.5 mg/kg, 0.25 mg/kg sodium sulfide, or vehicle twice daily for 14 days following the induction of limb ischemia. Laser doppler measurements of blood flow were taken pre- and post-ligation of the femoral artery on day 0, and then once daily on days 1, 3, 5, 7, 10, 12, and 14. All measurements were made with both the fine needle flow probe used in the pilot study, as well as a miniature surface probe. Hindlimb perfusion was also measured on day 14 by contrast ultrasound.

**Figure 3:**
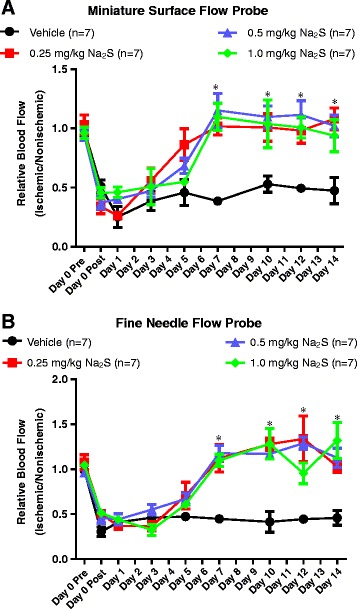
Twice Daily Administration of Na_2_S Enhances Ischemic Limb Perfusion at Doses as Low as 0.25 mg/kg. Panels **A** and **B** illustrate laser doppler blood flow measurements in the ischemic hindlimbs of rats given vehicle or Na_2_S (0.25, 0.5, or 1.0 mg/kg) i.v. twice daily for 14 days. Results in panels **A** and **B** were obtained with a miniature surface probe and a fine needle probe, respectively. * p < 0.05 vs Vehicle at respective time point by One Way ANOVA. Values are mean ± SEM.

Figure 3 illustrates the effects of twice daily administration of sulfide at these 3 doses on the recovery of blood flow in the ischemic hindlimb. Panels A and B display laser doppler measurements obtained with the miniature surface and fine needle flow probes, respectively. These results demonstrate that sodium sulfide at 0.25, 0.5, and 1.0 mg/kg doses significantly enhanced blood flow to the ischemic hindlimb by day 7, and this elevation was maintained through day 14. These results were consistent with those of the initial pilot, and suggested a 4-fold lower efficacious dose.

**Figure 4:**
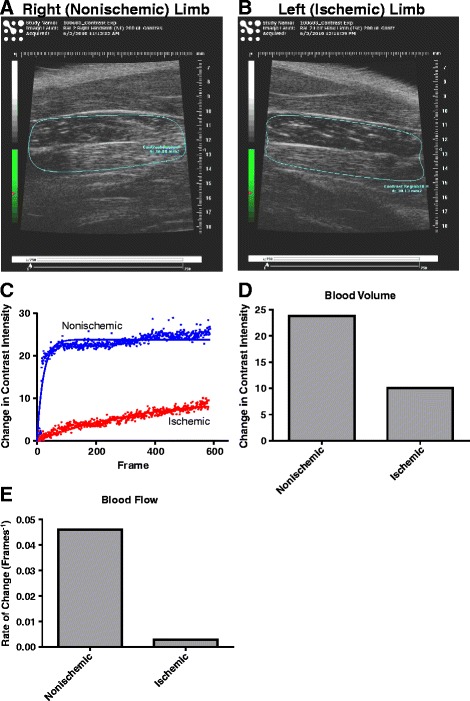
Contrast Ultrasound Measurements of Hind Limb Perfusion. Panels **A** and **B** are images of the gastrocnemius muscles in the nonischemic and ischemic limbs, respectively, from a single animal minutes after left femoral artery ligation. After microbubble injection, the change in contrast over time can be measured over a manually selected region of interest (ROI) within the image (shown enclosed by a blue line). Panel **C** is a scatter plot of the change in contrast intensity over time derived from the ROIs in panels **A** and **B**. Panels **D** and **E** demonstrate the cumulative changes in contrast intensity and the rates of change in intensity, respectively, which are derived from the best fit curves for the scatter plots in panel **C**.

In an effort to corroborate these laser doppler flow results, contrast enhanced ultrasound measurements of hindlimb skeletal muscle perfusion were used as an adjunct means of measurement. Figure 4 illustrates the images and data set obtained from a single animal on day 0 immediately following ischemic insult. Panels A and B are images of sagittal sections of the right (nonischemic) and left (ischemic) gastrocnemius muscles, respectively, of the same rat obtained minutes after left femoral artery ligation. Once satisfactory images were obtained, a solution of 3.0x10^8^ microbubbles was administered as an i.v. bolus, and the change in contrast over time was examined. Within a manually defined region of interest (enclosed by the blue outline in panels A and B), the change in contrast intensity was calculated for each frame of captured video and used to create the scatter plot shown in panel C. From the fitted curve of the scatter plot, the plateau and the slope can be calculated. The plateau represents the maximum change in contrast and is an indicator of blood volume, while the slope indicates the rate of change in contrast intensity and reflects the rate of blood flow. Panels D and E are representative illustrations of the changes in blood volume and flow rate, respectively, in the ischemic hindlimb just minutes after femoral artery ligation (generated from the curves in panel C). Total blood volume in the ischemic gastrocnemius of the rodent was decreased to 42% of the volume in the nonischemic limb, consistent with LD flow measurements taken just after the induction of ischemia. Panel E demonstrates a reduction in the rate of blood flow in the ischemic gastrocnemius of greater than 90% compared to the nonischemic muscle just after surgery.

**Figure 5:**
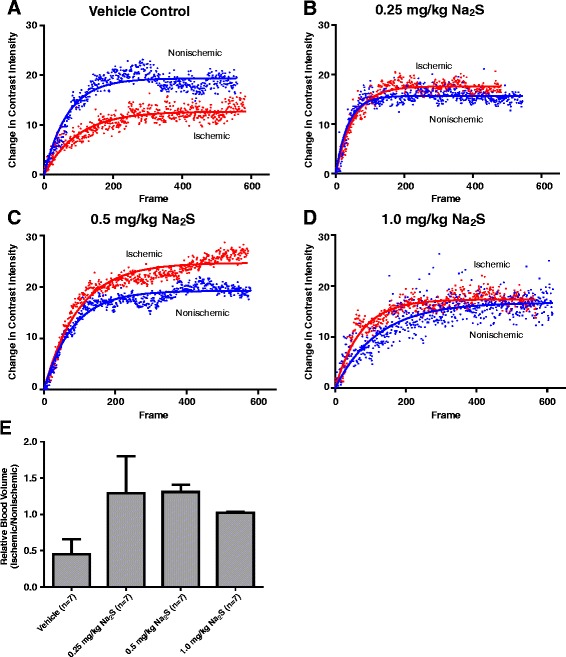
Contrast Ultrasound Perfusion Measurements Corroborate Laser Doppler Measurements. Panels **A**-**D** are representative scatter plots of changes in contrast in the ischemic and nonischemic hindlimbs of a single animal from vehicle, 0.25 mg/kg, 0.5 mg/kg, or 1.0 mg/kg Na_2_S treated cohorts, respectively, after 14 days of treatment. Panel **E** is a comparison of the change in contrast intensity in the ischemic limbs between treatment groups.

Figure 5 shows day 14 contrast ultrasound measurements of hindlimb perfusion from the same group of animals from Figure 3. Panels A-D are representative plots of contrast measurements taken from vehicle, 0.25 mg/kg, 0.5 mg/kg, or 1.0 mg/kg Na_a_S treated rats, respectively, suggesting that sulfide treatment at all 3 doses restores blood volume in the ischemic hindlimb by day 14. Panel E illustrates these results, which paralleled those obtained with both laser doppler flow probes on day 14 (Figures 3A and B).

### Experiment 3

A final experiment was designed to determine if the changes in blood flow with Na_2_S treatment persisted after treatment stopped, as well as to attempt to further elucidate a minimum effective dose. To address these questions, 32 animals were divided into 4 groups receiving 0.25 mg/kg sodium sulfide twice daily for 7 days (n = 8), 0.25 mg/kg twice daily for 3 days (n = 8), 0.5 mg/kg once a day for 7 days (n = 8), or vehicle twice daily for 7 days (n = 8). For this experiment, LD blood flow measurements were made pre- and post- ligation on day 0, and then on days 7, 14, and 28. Contrast ultrasound measurements were made on days 14 and 28.

**Figure 6:**
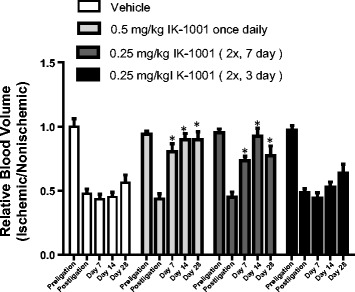
7 Day Administration of Na_2_S is Sufficient to Enhance Blood Flow to the Ischemic Hind Limb, which Persists 3 Weeks After Treatment is Stopped. This graph illustrates day 0, 7, 14, and 28 laser doppler blood flow measurements from the ischemic hind limbs of rats treated with either vehicle or Na_2_S at 0.25 mg/kg twice daily for 3 days, 0.25 mg/kg twice daily for 7 days, or 0.5 mg/kg once daily for 7 days. * p < 0.05 vs Postligation within the same treatment group by One Way ANOVA. Values are mean ± SEM.

Figure 6 illustrates the results of laser doppler measurements of blood flow from these animals. In animals that were treated with sodium sulfide at 0.25 mg/kg twice daily for 7 days or at 0.5 mg/kg once a day for 7 days, blood flow in the ischemic limb was significantly elevated by day 7. The results of the former treatment group are consistent with laser doppler results from the previous experiment, and the results from rats administered Na_2_S at 0.5 mg/kg suggest that multiple daily administrations are unnecessary. Furthermore, the elevation of blood flow at day 7 was maintained through day 28 in both treatment groups, suggesting that the positive effects of sulfide administration were not transient. Blood flow in the ischemic limbs of animals receiving 0.25 mg/kg twice a day for 3 days was never significantly different from vehicle treated animals at any time point.

**Figure 7:**
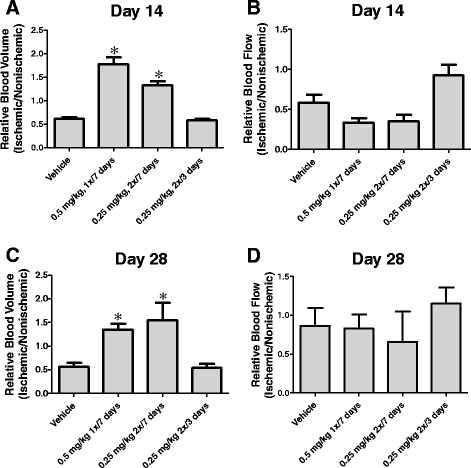
Corroboration of Laser Doppler Measurements with Ultrasound. Panels **A** and **B** are contrast ultrasound measurements of relative blood volume and blood flow rate, respectively, at day 14 from the ischemic hindlimbs of rats treated with either vehicle or Na_2_S at 0.25 mg/kg twice daily for 3 days, 0.25 mg/kg twice daily for 7 days, or 0.5 mg/kg once daily for 7 days. Panels **C** and **D** show relative blood volume and flow rate at day 28 from the same animals. * p < 0.05 vs Vehicle by One Way ANOVA. Values are mean ± SEM.

Contrast ultrasound was again used as an alternative means of assessing perfusion in the ischemic hindlimb, this time taking measurements at both day 14 and day 28. Consistent with laser doppler blood flow results, sodium sulfide administered twice daily at 0.25 mg/kg or once daily at 0.5 mg/kg for 7 days significantly elevated the total blood volume delivered to ischemic skeletal muscle. This effect was seen in both treatment groups at day 14 (Figure 7A) and day 28 (Figure 7C). While there was a positive impact on blood volume, there was no significant effect on the rate of blood flow (Day 14 – Figure 7B, Day 28 – Figure 7D).

## Discussion

In previous reports of the efficacy of sulfide in the hindlimb model, investigators utilized extended treatment periods. Wang et al. treated rats once daily for 4 weeks, while Bir et al. treated mice twice daily for 2 weeks. In the present study, we started with the dosing regimen used by Bir et al. and incrementally decreased the dosing concentration and frequency until we approached an MED. In our hands, we find that sodium sulfide administered intravenously at 0.25 mg/kg twice daily, or at 0.5 mg/kg once daily, for 7 days was sufficient to restore perfusion to ischemic skeletal muscle. Furthermore, this MED was also shown to provide a prolonged effect that was demonstrable at day 28.

In considering techniques for measuring tissue perfusion, intravenous injection of fluorescent microspheres is a cost effective approach, but it is an end point measurement; lacking the capacity for multiple measurements in a single animal. Laser doppler flowmetry affords the ability to noninvasively measure blood flow in the same animal repeatedly. However, shallow penetration allows only very superficial blood flow to be measured, and occlusion of subdermal microvascular blood flow with the probe itself is a concern with non-scanning probes like those used in this study. We therefore used contrast enhanced ultrasound in conjunction with laser doppler measurements. Ultrasound provides an actual image of the tissue that perfusion is being measured in, offers a much better depth of measurement, and circumvents issues with superficial microvascular occlusion. Results of contrast ultrasound perfusion measurements corroborated those using LD flowmetry.

Clinical trials of therapeutic angiogenesis for PAD and CLI have focused on delivery of constructs encoding genes for proangiogenic growth factors like VEGF, FGF, and HGF or certain bone marrow derived progenitor cell populations, with mixed results. The small size of hydrogen sulfide gas, along with its partition coefficient and rapid diffusion in and through lipid membranes [29,30], can facilitate its delivery to ischemic tissue despite a diminished or absent blood flow. At physiologic pH (7.4), sulfide exists predominantly as the more hydrophilic hydrosulfide anion (HS^−^), which would be subject to partitioning and is more sensitive to oxidation relative to its fully protonated counterpart. Sulfide becomes increasingly protonated as pH falls, reaching its pKa at 7.0, and then exists predominantly as H_2_S gas at more acidic pH. The acidification of ischemic tissue would only favor sulfide protonation, which should facilitate its delivery within ischemic tissue.

## Conclusions

The results of this study corroborate findings from other labs demonstrating that hydrogen sulfide enhances blood flow and vascular density in the ischemic rat hindlimb when administered at 0.5 and 1.0 mg/kg twice daily over 14 days. The minimum efficacious dose is either 0.25 mg/kg twice daily or 0.5 mg/kg once daily for 7 days. Shortening the treatment window to 3 days was noneffective. Furthermore, these effects appear to be long-lived. These data, together with published results from a number of other labs, lend further credence to the notion that hydrogen sulfide can enhance the vascularity and perfusion of ischemic tissue.

## Abbreviations

PAD: Peripheral arterial disease
CLI: Critical limb ischemia
MED: Minimum effective dose
ROS: Reactive oxygen species
VEGF: Vascular endothelial growth factor
HIF-1α: Hypoxia inducible factor 1 alpha
VEGFR2: Vascular endothelial growth factor receptor 2
HLI: Hindlimb ischemia
JVC: Jugular vein catheter
C: Celsius
PECAM-1: Platelet endothelial cell adhesion molecule 1
FGF: Fibroblast growth factor
HGF: Hepatocyte growth factor
H_2_S: Hydrogen sulfide
Na_2_S: Sodium sulfide
NaHS: Sodium hydrogen sulfide
ROI: Region of interest

## References

[1] Association TAH About Peripheral Artery Disease (PAD). 2014. Available from: http://www.heart.org/HEARTORG/Conditions/More/PeripheralArteryDisease/About-Peripheral-Artery-Disease-PAD_UCM_301301_Article.jsp.

[2] Varu VN, Hogg ME, Kibbe MR (2010). Critical limb ischemia. J Vasc Surg.

[3] Anderson JL, Halperin JL, Albert NM, Bozkurt B, Brindis RG, Curtis LH (2013). Management of patients with peripheral artery disease (compilation of 2005 and 2011 ACCF/AHA guideline recommendations): a report of the American College of Cardiology Foundation/American Heart Association Task Force on Practice Guidelines. Circulation.

[4] Bhatia M (2005). Hydrogen sulfide as a vasodilator. IUBMB Life.

[5] Papapetropoulos A, Pyriochou A, Altaany Z, Yang G, Marazioti A, Zhou Z (2009). Hydrogen sulfide is an endogenous stimulator of angiogenesis. Proc Natl Acad Sci U S A.

[6] Stein A, Bailey SM (2013). Redox Biology of hydrogen sulfide: implications for physiology, pathophysiology, and pharmacology. Redox Biol.

[7] Calvert JW, Elston M, Nicholson CK, Gundewar S, Jha S, Elrod JW (2010). Genetic and pharmacologic hydrogen sulfide therapy attenuates ischemia-induced heart failure in mice. Circulation.

[8] Kondo K, Bhushan S, King AL, Prabhu SD, Hamid T, Koenig S (2013). H(2)S protects against pressure overload-induced heart failure via upregulation of endothelial nitric oxide synthase. Circulation.

[9] Polhemus DJ, Kondo K, Bhushan S, Bir SC, Kevil CG, Murohara T (2013). Hydrogen sulfide attenuates cardiac dysfunction after heart failure via induction of angiogenesis. Circ Heart Fail.

[10] Chunyu Z, Junbao D, Dingfang B, Hui Y, Xiuying T, Chaoshu T (2003). The regulatory effect of hydrogen sulfide on hypoxic pulmonary hypertension in rats. Biochem Biophys Res Commun.

[11] Yan H, Du J, Tang C (2004). The possible role of hydrogen sulfide on the pathogenesis of spontaneous hypertension in rats. Biochem Biophys Res Commun.

[12] Wang Y, Zhao X, Jin H, Wei H, Li W, Bu D (2009). Role of hydrogen sulfide in the development of atherosclerotic lesions in apolipoprotein E knockout mice. Arterioscler Thromb Vasc Biol.

[13] Qiao W, Chaoshu T, Hongfang J, Junbao D (2010). Endogenous hydrogen sulfide is involved in the pathogenesis of atherosclerosis. Biochem Biophys Res Commun.

[14] Nicholson CK, Calvert JW (2010). Hydrogen sulfide and ischemia-reperfusion injury. Pharmacol Res.

[15] King AL, Lefer DJ (2011). Cytoprotective actions of hydrogen sulfide in ischaemia-reperfusion injury. Exp Physiol.

[16] Mustafa AK, Gadalla MM, Sen N, Kim S, Mu W, Gazi SK (2009). H2S signals through protein S-sulfhydration. Sci Signal.

[17] Sen N, Paul BD, Gadalla MM, Mustafa AK, Sen T, Xu R (2012). Hydrogen sulfide-linked sulfhydration of NF-kappaB mediates its antiapoptotic actions. Mol Cell.

[18] Yang G, Zhao K, Ju Y, Mani S, Cao Q, Puukila S (2013). Hydrogen sulfide protects against cellular senescence via S-sulfhydration of Keap1 and activation of Nrf2. Antioxid Redox Signal.

[19] Nicholls P, Kim JK (1981). Oxidation of sulphide by cytochrome aa3. Biochim Biophys Acta.

[20] Nicholls P, Kim JK (1982). Sulphide as an inhibitor and electron donor for the cytochrome c oxidase system. Can J Biochem.

[21] Kolluru GK, Shen X, Bir SC, Kevil CG (2013). Hydrogen sulfide chemical biology: pathophysiological roles and detection. Nitric Oxide.

[22] Cai WJ, Wang MJ, Moore PK, Jin HM, Yao T, Zhu YC (2007). The novel proangiogenic effect of hydrogen sulfide is dependent on Akt phosphorylation. Cardiovasc Res.

[23] Qipshidze N, Metreveli N, Mishra PK, Lominadze D, Tyagi SC (2012). Hydrogen sulfide mitigates cardiac remodeling during myocardial infarction via improvement of angiogenesis. Int J Biol Sci.

[24] Predmore BL, Julian D, Cardounel AJ (2011). Hydrogen sulfide increases nitric oxide production from endothelial cells by an akt-dependent mechanism. Front Physiol.

[25] Bir SC, Kolluru GK, McCarthy P, Shen X, Pardue S, Pattillo CB (2012). Hydrogen sulfide stimulates ischemic vascular remodeling through nitric oxide synthase and nitrite reduction activity regulating hypoxia-inducible factor-1alpha and vascular endothelial growth factor-dependent angiogenesis. J Am Heart Assoc.

[26] Liu X, Pan L, Zhuo Y, Gong Q, Rose P, Zhu Y (2010). Hypoxia-inducible factor-1alpha is involved in the pro-angiogenic effect of hydrogen sulfide under hypoxic stress. Biol Pharm Bull.

[27] Tao BB, Liu SY, Zhang CC, Fu W, Cai WJ, Wang Y (2013). VEGFR2 functions as an H2S-targeting receptor protein kinase with its novel Cys1045-Cys1024 disulfide bond serving as a specific molecular switch for hydrogen sulfide actions in vascular endothelial cells. Antioxid Redox Signal.

[28] Wang MJ, Cai WJ, Li N, Ding YJ, Chen Y, Zhu YC (2010). The hydrogen sulfide donor NaHS promotes angiogenesis in a rat model of hind limb ischemia. Antioxid Redox Signal.

[29] Mathai JC, Missner A, Kugler P, Saparov SM, Zeidel ML, Lee JK (2009). No facilitator required for membrane transport of hydrogen sulfide. Proc Natl Acad Sci U S A.

[30] Cuevasanta E, Denicola A, Alvarez B, Moller MN (2012). Solubility and permeation of hydrogen sulfide in lipid membranes. PLoS One.

